# Social genetic effects for growth in pigs differ between boars and gilts

**DOI:** 10.1186/s12711-018-0375-0

**Published:** 2018-02-01

**Authors:** Hanne M. Nielsen, Birgitte Ask, Per Madsen

**Affiliations:** 1 Breeding and Genetics, SEGES, Pig Research Centre, Danish Agriculture and Food Council F.m.b.A., Axelborg, Axeltorv 3, 1609 Copenhagen V, Denmark; 2 Department of Molecular Biology and Genetics, Center for Quantitative Genetics and Genomics, Blichers Allé 20, 8830 Tjele, Denmark

## Abstract

**Background:**

Average daily gain (ADG) in pigs is affected by the so-called social (or indirect) genetic effects (SGE). However, SGE may differ between sexes because boars grow faster than gilts and their social behaviours differ. We hypothesized that direct genetic effects (DGE) and SGE for ADG in pigs differ between boars and gilts and that accounting for these differences will improve the predictive ability of a social genetic effects model (SGM). Our data consisted of ADG from 30 to 94 kg for 32,212 uncastrated males (boars) and 48,252 gilts that were raised in sex-specific pens. Data were analyzed using a univariate model with sex as a fixed effect and a bivariate model with ADG in boars and gilts as separate traits using both a classical animal model (CM) and a SGM.

**Results:**

With the univariate model, the heritability for ADG was 0.22 ± 0.01 for the CM, while the estimate of the total heritable variance (T^2^) was 0.23 ± 0.01 with the SGM. With the bivariate model, the genetic variance for SGE was higher for boars (13.8 ± 5.8) than for gilts (9.3 ± 3.9). For the bivariate model, T^2^ was 0.32 ± 0.02 for boars and 0.27 ± 0.01 for gilts. Estimates of the genetic correlations between DGE (0.88 ± 0.02) and SGE (0.30 ± 0.30) for boars versus gilts indicated that ADG in boars and gilts are different traits. Moreover, the estimate of the genetic correlation between DGE and SGE indicated presence of genetic effects of competition among gilts but not among boars. Compared to a CM, the univariate SGM improved predictive ability significantly only for gilts and the bivariate SGM improved predictive ability significantly for both boars and gilts.

**Conclusions:**

We found significant genetic variances of SGE for ADG. The covariance between DGE and SGE was much more negative for gilts than for boars when applying the bivariate model. Because the estimate of the genetic correlation for ADG between gilts and boars differed significantly from 1 and the predictive ability for boars and gilts was improved significantly with the bivariate model, we recommend the use of a bivariate model to estimate both SGE and DGE for ADG in pigs.

## Background

Daily gain is a key trait in pig breeding goals because it contributes to economic efficiency. Its estimated heritability is moderate but several studies have shown that the total heritable variation is higher that the heritability estimated from a classical model because this trait is affected by so-called social (or indirect) genetic effects (SGE) [1–3]. Thus, the daily gain of a pig depends both on the genes of the pig itself (direct genetic effects, DGE) and on the genes of the other pigs in the same group [1, 4, 5]. By including SGE in the model for prediction of breeding values, it should be possible to capture the total heritable variation in daily gain, which should lead to a greater rate of genetic response in daily gain. However, to date the social genetic model has not been shown to consistently improve predictive ability compared to the classical animal model [6]. There may be several explanations for this. First, it is more difficult to obtain accurate estimates of social breeding values compared to breeding values from a classical animal model because the accuracy of social breeding values depends on the number of phenotyped offspring per sire, the number of groups in which the sires have offspring, and the group size [6, 7]. Second, a large number of groups is needed to estimate genetic variance components accurately, especially to estimate the genetic correlation between SGE and DGE [8]. Finally, the fixed effects that are fitted in the model may themselves have a social genetic background, which to date has not been properly accounted for when estimating variance components for social genetic effects models. For example, when analyzing growth traits, sex is usually fitted as a fixed effect. However, social genetic variances may differ between sexes because boars grow faster than gilts [9]. Difference in variance components between sexes could reduce predictive ability of the social genetic model if these are not accounted for.

Social genetic effects may also differ between sexes due to differences in social behaviour. For example, on the one hand, there are indications that female pigs are more likely to tail bite than male pigs [10] and that female pigs have less tail damage than castrated pigs [11]. On the other hand, aggressive behavior and mounting behavior are more frequent in boars than in gilts [12, 13]. Because of these possible differences in social behavior between male and female pigs, it is likely that SGE also differ between sexes but this has not been investigated to date. If SGE differ between males and females, this must be accounted for in the estimation of breeding values. Therefore, we hypothesize that DGE and SGE for daily gain in pigs differ between boars and gilts and that accounting for these differences will improve the predictive ability of a social genetic effects model.

## Methods

### Ethics statement

The data used in the current study were part of routine recordings of pigs in performance tests in the nucleus herds of the Danish pig breeding program, DanAvl. Only weight records were used and these require no ethical approval.

### Pigs and housing

Data used in this study were routinely collected records from pigs that enter the performance test in the nucleus herds of the Danish pig breeding program, DanAvl. The pigs were purebred Danish Landrace pigs raised in 13 herds in Denmark. After weaning, the pigs were housed in weaner pig units. Before reaching 28 kg, the pigs were moved to slaughter pig pens. The strategy on how pigs were mixed in the slaughter pig pens differed between farms, and on some farms, pigs were less likely to be mixed with other pigs from the same litter than on other farms. Females (gilts) and males were kept in separate pens but in the same barn except for two farms, where males and females were in different barns. All male pigs were uncastrated (boars). Boars were on average 82.5 ± 8.4 days old and weighed 30.3 ± 1.9 kg when they entered the performance test in the slaughter pig pens (Table 1). The average age and average weight of gilts when entering the performance test were 81.6 ± 8.2 days and 30.4 ± 1.9 kg, respectively. Pigs were performance-tested for growth until their average weight reached 94 kg. Pigs were fed *ad libitum* with a dry feed during the test. Group sizes at the start of the performance test varied from 8 to 15 pigs per pen and the space allowance per pig was 0.75 to 1.0 m^2^ at the start of the performance test. At the end of the test, all pigs in a given pen were weighed to the nearest kg. If a pig left the pen because of death or illness, the date and reason for leaving the pen were recorded. No pigs were allowed to re-enter their pen after having been removed, and no pigs were allowed to change between pens once the performance test had started.

**Table 1:** Descriptive statistics for boars and gilts

Variable (units)	Boars		Gilts	
	Mean	SD	Mean	SD
Number of animals	32,212		48,252	
Number of observations	30,447		45,942	
Number of groups	2864		4445	
Number of incomplete groups	1765		2310	
Number of compartments	789		789	
Number of litters	8611		10,209	
Average group size	11.7		11.3	
Average genetic relationship between pigs within a group	0.183		0.182	
Age at start of test (days)	82.5	8.4	81.6	8.2
Age at end of test (days)	143.9	12.4	145.9	12.1
Weight at start of test (g)	30.3	1.9	30.4	1.9
Weight at end of test (g)	94.1	9.7	94.0	8.8
Average daily gain (g)	1031.1	137.5	977.3	115.2

SD: standard deviation

### Data

Data consisted of 80,464 pigs but only 76,389 pigs had a weight record at the end of the test. Thus, 4075 pigs were dead or sold or removed due to illness before the end of the performance test. Average daily gain (ADG) was calculated as $\frac{{{\text{WEIGHT}}_{\text{end}} - {\text{WEIGHT}}_{\text{start}} }}{\text{DAYS}},$ where ${\text{WEIGHT}}_{\text{end}}$ and ${\text{WEIGHT}}_{\text{start}}$ are the weights of the pig at the end and the start of the performance test, and DAYS is the duration of the performance test in days.

Complete pedigrees were traced six generations back and included a total of 1142 sires and 11,369 dams. Data were edited by excluding compartments that had less than five pens. The compartment was a part of the barn in which pigs finished the performance test within the same time period.

### Descriptive statistics

Overall, there were 32,212 boars in 2864 groups and 48,252 gilts in 4445 groups (Table 1). The number of boars was smaller than that of gilts because smaller numbers of boars were tested on the breeding farms. In addition, around 6% of all tested boars were moved from the herds to a test station for performance testing. In total, there were 789 compartments and the number of groups per compartment ranged from 5 to 25. For boars, 5.5% of the pigs did not finish the performance test because of sales, death, or illness, whereas 4.8% of the gilts did not complete the performance test. This resulted in 61.2% incomplete groups of boars and 52% incomplete groups of gilts. The ADG was higher for boars (1031.1 g) than for gilts (977.3 g). Group sizes at the start of the performance test ranged from 8 to 15 for both boars and gilts and were on average 11.3 for boars and 11.7 for gilts. The average additive genetic relationship among pigs within a group was 0.18 for both boars and gilts.

### Estimation of genetic parameters

Variance components were estimated by using both a univariate model, in which sex was treated as a fixed effect, and a bivariate model, in which ADG was treated as a separate trait in boars and gilts. A classical animal model and a social genetic effects model were fitted for both the univariate and the bivariate model.

The classical univariate model was:

1 $${\mathbf{y}} = {\mathbf{Xb}} + {\mathbf{Z}}_{\text{D}} {\mathbf{a}}_{\text{D}} + {\mathbf{Z}}_{\text{l}} {\mathbf{l}} + {\mathbf{Z}}_{\text{g}} {\mathbf{g}} + {\mathbf{e}},$$

where **y** = ADG during the performance test, **b** is a vector of covariates of age and age squared at the start of the performance test nested within sex, as well as the fixed effects of sex (boar or gilt) and compartment. Furthermore, **a**_D_ is a vector of random direct additive genetic effects, **l** is a vector of random litter effects, **g** is a vector of random group effects, and **e** is a vector of residuals. **X**, **Z**_D_, **Z**_1_ and **Z**_g_ are incidence matrices. Assumptions for random effects were:

$$\left[ {\begin{array}{*{20}c} {\begin{array}{*{20}c} {{\mathbf{a}}_{{\mathbf{D}}} } \\ {\mathbf{l}} \\ {\mathbf{g}} \\ \end{array} } \\ {\mathbf{e}} \\ \end{array} } \right]\sim\,N\left( {\left[ {\begin{array}{*{20}c} {\begin{array}{*{20}c} {\bf 0} \\ {\bf 0} \\ {\bf 0} \\ \end{array} } \\ {\bf 0} \\ \end{array} } \right],\left[ {\begin{array}{*{20}c} {\begin{array}{*{20}c} {\upsigma_{{{\mathbf{A}}_{\text{D}} }}^{2} {\mathbf{A}}} \\ {\bf 0} \\ {\bf 0} \\ \end{array} } \\ {\bf 0} \\ \end{array} \begin{array}{*{20}c} {\begin{array}{*{20}c} {\bf 0} \\ {\upsigma_{\text{l}}^{2} {\mathbf{I}}_{\text{l}} } \\ {\bf 0} \\ \end{array} } \\ {\bf 0} \\ \end{array} \begin{array}{*{20}c} {\begin{array}{*{20}c} {\bf 0} \\ {\bf 0} \\ {\upsigma_{\text{g}}^{2} {\mathbf{I}}_{\text{g}} } \\ \end{array} } \\ {\bf 0} \\ \end{array} \begin{array}{*{20}c} {\begin{array}{*{20}c} {\bf 0} \\ {\bf 0} \\ {\bf 0} \\ \end{array} } \\ {\upsigma_{\text{e}}^{2} {\mathbf{I}}_{\text{e}} } \\ \end{array} } \right]} \right),$$

where **A** is the additive relationship matrix, $\upsigma_{{{\mathbf{A}}{\text{D}}}}^{2}$ is the direct additive genetic variance, $\upsigma_{\text{l}}^{2}$ is the variance of the litters in which the pigs were born, $\upsigma_{\text{g}}^{2}$ is the variance of the groups in which the pigs were penned during the performance test, $\upsigma_{e}^{2}$ is the residual variance, **I**_l_, **I**_g_, and **I**_e_ are identity matrices of dimensions equal to the number of groups, number of litters, and number of observations, respectively.

The univariate social genetic effects model was [14]:

2 $${\mathbf{y}} = {\mathbf{Xb}} + {\mathbf{Z}}_{\text{D}} {\mathbf{a}}_{\text{D}} + {\mathbf{Z}}_{\text{S}} {\mathbf{a}}_{\text{S}} + {\mathbf{Z}}_{\text{l}} {\mathbf{l}} + {\mathbf{Z}}_{\text{g}} {\mathbf{g}} + {\mathbf{e}},$$

where **a**_S_ is a vector of random social additive genetic effects and all other variables are as described for Model 1. Assumptions for the genetic effects **a**_D_ and **a**_S_ were:

$$\left[ {\begin{array}{*{20}c} {{\mathbf{a}}_{\text{D}} } \\ {{\mathbf{a}}_{\text{S}} } \\ \end{array} } \right]\sim\,N\left( {\left[ {\begin{array}{*{20}c} {\bf 0} \\ {\bf 0} \\ \end{array} } \right],\left[ {\begin{array}{*{20}c} {\upsigma_{{{\mathbf{A}}_{\text{D}} }}^{2} } \\ {\upsigma_{{{\mathbf{A}}_{\text{DS}} }} } \\ \end{array} \begin{array}{*{20}c} {\upsigma_{{{\mathbf{A}}_{\text{DS}} }} } \\ {\upsigma_{{{\mathbf{A}}_{\text{S}} }}^{2} } \\ \end{array} } \right] \otimes {\mathbf{A}}} \right),$$

where $\upsigma_{{{\mathbf{A}}{\text{S}}}}^{2}$ is the social additive genetic variance and $\upsigma_{{{\mathbf{A}}_{\text{DS}} }}$ is the direct-social additive genetic covariance. Assumptions for the other random effects were the same as for Model (1). The group effect accounted for correlated residuals between group mates [15].

Except for the fixed effect of sex, the bivariate classical model included the same fixed and random effects as the univariate model (Model 1). Subscripts *b* and *g* refer to boars and gilts such that **y**_*b*_ is ADG for boars and **y**_*g*_ is ADG for gilts.

3 $$\left[ {\begin{array}{*{20}c} {{\mathbf{y}}_{b} } \\ {{\mathbf{y}}_{g} } \\ \end{array} } \right] = \left[ {\begin{array}{*{20}c} {{\mathbf{X}}_{b} } & 0 \\ 0 & {{\mathbf{X}}_{g} } \\ \end{array} } \right]\left[ {\begin{array}{*{20}c} {{\mathbf{b}}_{b} } \\ {{\mathbf{b}}_{g} } \\ \end{array} } \right] + \left[ {\begin{array}{*{20}c} {{\mathbf{Z}}_{{{\text{D}}b}} } & {\bf 0} \\ {\bf 0} & {{\mathbf{Z}}_{{{\mathbf{D}}g}} } \\ \end{array} } \right]\left[ {\begin{array}{*{20}c} {{\mathbf{a}}_{{{\text{D}}b}} } \\ {{\mathbf{a}}_{{{\text{D}}g}} } \\ \end{array} } \right] + \left[ {\begin{array}{*{20}c} {{\mathbf{Z}}_{{{\mathbf{l}}b}} } & {\bf 0} \\ {\bf 0} & {{\mathbf{Z}}_{{{\mathbf{l}}g}} } \\ \end{array} } \right]\left[ {\begin{array}{*{20}c} {{\mathbf{l}}_{b} } \\ {{\mathbf{l}}_{g} } \\ \end{array} } \right] + \left[ {\begin{array}{*{20}c} {{\mathbf{Z}}_{{{\mathbf{g}}b}} } & {\bf 0} \\ {\bf 0} & {{\mathbf{Z}}_{{{\mathbf{g}}g}} } \\ \end{array} } \right]\left[ {\begin{array}{*{20}c} {{\mathbf{g}}_{b} } \\ {{\mathbf{g}}_{g} } \\ \end{array} } \right] + \left[ {\begin{array}{*{20}c} {{\mathbf{e}}_{b} } \\ {{\mathbf{e}}_{g} } \\ \end{array} } \right].$$

Because records were nested within group and sex, there were no co-variances between group effects for boars and gilts or co-variances between residuals for boars and gilts, and assumptions for the random effects were:

$$\left[ {\begin{array}{*{20}c} {\begin{array}{*{20}c} {\left[ {\begin{array}{*{20}c} {{\mathbf{a}}_{{{\text{D}}b}} } \\ {{\mathbf{a}}_{{{\text{D}}g}} } \\ \end{array} } \right]} \\ {\left[ {\begin{array}{*{20}c} {{\mathbf{l}}_{b} } \\ {{\mathbf{l}}_{g} } \\ \end{array} } \right]} \\ {\left[ {\begin{array}{*{20}c} {{\mathbf{g}}_{b} } \\ {{\mathbf{g}}_{g} } \\ \end{array} } \right]} \\ \end{array} } \\ {\left[ {\begin{array}{*{20}c} {{\mathbf{e}}_{b} } \\ {{\mathbf{e}}_{g} } \\ \end{array} } \right]} \\ \end{array} } \right]\sim\,N\left( {\left[ {\begin{array}{*{20}c} {\begin{array}{*{20}c} {\left[ {\begin{array}{*{20}c} {\bf 0} \\ {\bf 0} \\ \end{array} } \right]} \\ {\left[ {\begin{array}{*{20}c} {\bf 0} \\ {\bf 0} \\ \end{array} } \right]} \\ {\left[ {\begin{array}{*{20}c} {\bf 0} \\ {\bf 0} \\ \end{array} } \right]} \\ \end{array} } \\ {\left[ {\begin{array}{*{20}c} {\bf 0} \\ {\bf 0} \\ \end{array} } \right]} \\ \end{array} } \right],\left[ {\begin{array}{*{20}c} {\begin{array}{*{20}c} {\left[ {\begin{array}{*{20}c} {\upsigma_{{{\mathbf{A}}_{{{\text{D}}b}} }}^{2} } \\ {\upsigma_{{{\mathbf{A}}_{{{\text{D}}b{\text{D}}g}} }} } \\ \end{array} \begin{array}{*{20}c} {\upsigma_{{{\mathbf{A}}_{{{\text{D}}b{\text{D}}g}} }} } \\ {\upsigma_{{{\mathbf{A}}_{{{\text{D}}g}} }}^{2} } \\ \end{array} } \right] \otimes {\mathbf{A}}} \\ {\bf 0} \\ {\bf 0} \\ \end{array} } \\ {\bf 0} \\ \end{array} \begin{array}{*{20}c} {\begin{array}{*{20}c} {\bf 0} \\ {\left[ {\begin{array}{*{20}c} {\upsigma_{{{\mathbf{l}}b}}^{2} } \\ {\upsigma_{{{\mathbf{l}}bg}} } \\ \end{array} \begin{array}{*{20}c} {\upsigma_{{{\mathbf{l}}bg}} } \\ {\upsigma_{{{\mathbf{l}}g}}^{2} } \\ \end{array} } \right] \otimes {\mathbf{I}}_{{\mathbf{l}}} } \\ {\bf 0} \\ \end{array} } \\ {\bf 0} \\ \end{array} \begin{array}{*{20}c} {\begin{array}{*{20}c} {\bf 0} \\ {\bf 0} \\ {\left[ {\begin{array}{*{20}c} {\upsigma_{{{\mathbf{g}}b}}^{2} } \\ {\bf 0} \\ \end{array} \begin{array}{*{20}c} {\bf 0} \\ {\upsigma_{{{\mathbf{g}}g}}^{2} } \\ \end{array} } \right] \otimes {\mathbf{I}}_{{\mathbf{g}}} } \\ \end{array} } \\ {\bf 0} \\ \end{array} \begin{array}{*{20}c} {\begin{array}{*{20}c} {\bf 0} \\ {\bf 0} \\ {\bf 0} \\ \end{array} } \\ {\left[ {\begin{array}{*{20}c} {\upsigma_{{{\mathbf{e}}b}}^{2} } \\ {\bf 0} \\ \end{array} \begin{array}{*{20}c} 0 \\ {\upsigma_{{{\mathbf{e}}g}}^{2} } \\ \end{array} } \right] \otimes {\mathbf{I}}_{{\mathbf{e}}} } \\ \end{array} } \right]} \right),$$

where **a**_D*b*_ and **a**_D*g*_ are DGE, **l**_*b*_ and **l**_*g*_ are the litter effects, **g**_*b*_ and **g**_*g*_ are group effects, and **e**_*b*_ and **e**_*g*_ are residuals for boars and gilts, respectively.

The bivariate social genetic effects model was:

$$\left[ {\begin{array}{*{20}c} {{\mathbf{y}}_{b} } \\ {{\mathbf{y}}_{g} } \\ \end{array} } \right] = \left[ {\begin{array}{*{20}c} {{\mathbf{X}}_{b} } & {\bf 0} \\ {\bf 0} & {{\mathbf{X}}_{g} } \\ \end{array} } \right]\left[ {\begin{array}{*{20}c} {{\mathbf{b}}_{b} } \\ {{\mathbf{b}}_{g} } \\ \end{array} } \right] + \left[ {\begin{array}{*{20}c} {{\mathbf{Z}}_{{{\text{D}}b}} } & {\bf 0} \\ {\bf 0} & {{\mathbf{Z}}_{{{\text{D}}g}} } \\ \end{array} } \right]\left[ {\begin{array}{*{20}c} {{\mathbf{a}}_{{{\text{D}}b}} } \\ {{\mathbf{a}}_{{{\text{D}}g}} } \\ \end{array} } \right]$$

$$+ \left[ {\begin{array}{*{20}c} {{\mathbf{Z}}_{{{\text{S}}b}} } & {\bf 0} \\ {\bf 0} & {{\mathbf{Z}}_{{{\text{S}}g}} } \\ \end{array} } \right]\left[ {\begin{array}{*{20}c} {{\mathbf{a}}_{{{\text{S}}b}} } \\ {{\mathbf{a}}_{{{\text{S}}g}} } \\ \end{array} } \right] + \left[ {\begin{array}{*{20}c} {{\mathbf{Z}}_{{{\mathbf{l}}b}} } & {\bf 0} \\ {\bf 0} & {{\mathbf{Z}}_{{{\mathbf{l}}g}} } \\ \end{array} } \right]\left[ {\begin{array}{*{20}c} {{\mathbf{l}}_{b} } \\ {{\mathbf{l}}_{g} } \\ \end{array} } \right]$$

4 $$+ \left[ {\begin{array}{*{20}c} {{\mathbf{Z}}_{{{\mathbf{g}}b}} } & {\bf 0} \\ {\bf 0} & {{\mathbf{Z}}_{{{\mathbf{g}}g}} } \\ \end{array} } \right]\left[ {\begin{array}{*{20}c} {{\mathbf{g}}_{b} } \\ {{\mathbf{g}}_{g} } \\ \end{array} } \right] + \left[ {\begin{array}{*{20}c} {{\mathbf{e}}_{b} } \\ {{\mathbf{e}}_{g} } \\ \end{array} } \right].$$

The covariance structures for litter (*i*), group (*g*), and residual were as in the classical bivariate Model (2), but for the genetic effects (direct and social), the covariance structure was:

$${\text{var}}\left[ {\begin{array}{*{20}c} {\begin{array}{*{20}c} {{\mathbf{a}}_{{{\text{D}}b}} } \\ {{\mathbf{a}}_{{{\text{D}}g}} } \\ {{\mathbf{a}}_{{{\text{S}}b}} } \\ \end{array} } \\ {{\mathbf{a}}_{{{\text{S}}g}} } \\ \end{array} } \right] = \left[ {\begin{array}{*{20}c} {\begin{array}{*{20}c} {{\upsigma}_{{{\mathbf{A}}_{{{\text{D}}b}} }}^{2} } \\ {{\upsigma}_{{{\mathbf{A}}_{{{\text{D}}b{\text{D}}g}} }} } \\ {{\upsigma}_{{{\mathbf{A}}_{{{\text{D}}b{\text{S}}b}} }} } \\ \end{array} } \\ {{\upsigma}_{{{\mathbf{A}}_{{{\text{D}}b{\text{S}}g}} }} } \\ \end{array} \begin{array}{*{20}c} {\begin{array}{*{20}c} {{\upsigma}_{{{\mathbf{A}}_{{{\text{D}}b{\text{D}}g}} }} } \\ {{\upsigma}_{{{\mathbf{A}}_{{{\text{D}}g}} }}^{2} } \\ {{\upsigma}_{{{\mathbf{A}}_{{{\text{D}}g{\text{S}}b}} }} } \\ \end{array} } \\ {{\upsigma}_{{{\mathbf{A}}_{{{\text{D}}g{\text{S}}g}} }} } \\ \end{array} \begin{array}{*{20}c} {\begin{array}{*{20}c} {{\upsigma}_{{{\mathbf{A}}_{{{\text{D}}b{\text{S}}b}} }} } \\ {{\upsigma}_{{{\mathbf{A}}_{{{\text{D}}g{\text{S}}b}} }} } \\ {{\upsigma}_{{{\mathbf{A}}_{{{\text{S}}b}} }}^{2} } \\ \end{array} } \\ {{\upsigma}_{{{\mathbf{A}}_{{{\text{S}}b{\text{S}}g}} }} } \\ \end{array} \begin{array}{*{20}c} {\begin{array}{*{20}c} {{\upsigma}_{{{\mathbf{A}}_{{{\text{D}}b{\text{S}}g}} }} } \\ {{\upsigma}_{{{\mathbf{A}}_{{{\text{D}}g{\text{S}}g}} }} } \\ {{\upsigma}_{{{\mathbf{A}}_{{{\text{S}}b{\text{S}}g}} }} } \\ \end{array} } \\ {{\upsigma}_{{{\mathbf{A}}_{{{\text{S}}g}} }}^{2} } \\ \end{array} } \right] \otimes {\mathbf{A}}.$$

All analyses were performed by AI-REML, using the DMU software [16]. For the social genetic effects models (Models 2 and 4), the total heritable variance, phenotypic variance, and total heritable variance relative to the phenotypic variance were calculated [1, 4].

The total heritable variance ($\upsigma_{TBV}^{2}$) was calculated as:

$$\upsigma_{TBV}^{2} =\upsigma_{{{\mathbf{A}}{\text{D}}}}^{2} + 2\left( {n - 1} \right)\upsigma_{{{\mathbf{A}}_{\text{DS}} }} + \left( {n - 1} \right)^{2}\upsigma_{{{\mathbf{A}}{\text{S}}}}^{2} ,$$

where *n* is the average number of pigs in each group at the start of the performance test.

The proportion of total heritable variance to the phenotypic variance, that determines the potential of the population to respond to selection (T^2^) [1, 4] was calculated as $\upsigma_{TBV}^{2} /\upsigma_{P}^{2}$, where $\upsigma_{P}^{2}$ is the phenotypic variance.

In our study, pigs within a group were related. Thus, $\upsigma_{P}^{2}$ was calculated for related individuals as [5]:

$$\upsigma_{P}^{2} =\upsigma_{{{\mathbf{A}}_{\text{D}} }}^{2} +\upsigma_{g}^{2} +\upsigma_{l}^{2} +\upsigma_{e}^{2} + \left( {n - 1} \right)r\left[ {2\upsigma_{{{\mathbf{A}}_{\text{DS}} }} + \left( {n - 2} \right)\upsigma_{{{\mathbf{A}}_{\text{S}} }}^{2} } \right],$$

where *r* is the average additive genetic relationship within groups.

To assess the importance of SGE, log likelihood tests were performed by calculating differences in likelihood between the univariate classical Model (1) and the univariate social genetic Model (2). Similarly, likelihoods of the bivariate classical Model (3) and the bivariate social genetic effects Model (4) were compared.

### Predictive ability

We compared the abilities of the univariate and bivariate models to predict phenotypes by calculating correlations between predicted breeding values and phenotypes corrected for fixed effects in a validation dataset. For this purpose, a dataset was constructed by omitting phenotypes recorded during the last half year (October 1st 2015 to April 1st 2016), which were used for validation. Using this data, breeding values were predicted for all animals in the validation dataset but correlations between predicted and corrected phenotypes were calculated separately for boars and gilts. The number of observations in the prediction dataset was 57,038 and the number of observations in the validation dataset was 23,426. The corrected phenotypes were the residuals from a GLM model in the SAS software version 9.4 (Copyright © 2002-2012 SAS Institute Inc. SAS) that included only the fixed effects described above for the univariate and bivariate models.

Four different predicted breeding values were evaluated [6]:

1. $$DBV_{{CLASSIC_{i} }} ,$$
2. $$DBV_{{SOCIAL_{i} }} ,$$
3. $$\sum SBV_{{SOCIAL_{j} }} ,$$
4. $$P_{{TOTAL_{i} }} = DBV_{{SOCIAL_{i} }} + \sum SBV_{{SOCIAL_{j} }} ,$$

where $DBV_{{CLASSIC_{i} }}$ and $DBV_{{SOCIAL_{i} }}$ are the direct breeding values for individual *i* from the classical model and the social genetic effects model, respectively, $\sum SBV_{{SOCIAL_{j} }}$ is the sum of the social breeding values of mates *j* from the social genetic effects model. $P_{{TOTAL_{i} }}$ is the total phenotype of an individual according to the social genetic effects model [4].

Correlations of predicted breeding values with corrected phenotypes were only computed for those animals for which their sires had a least one progeny with phenotype in both the prediction dataset and the validation dataset. Thus, the minimum and maximum numbers of offspring per boar were 1 and 101, respectively. Furthermore, only groups without missing phenotypes were included. Thus, the results on predictive ability of the models were based on 45 sires and 156 groups. For both the univariate and bivariate models, differences in predictive ability of the classical and social genetic effects models were evaluated by testing whether the correlation between the corrected phenotypes $\hat{P}_{i}$ and $DBV_{{CLASSIC_{i} }}$ were statistically different from the correlation between the corrected phenotypes $\hat{P}_{i}$ and $P_{{TOTAL_{i} }}$ using a Hotelling-Williams test [17].

## Results

### Univariate models with sex as a fixed effect

The univariate classical and social genetic effects models had similar estimates of variance components for DGE, group and litter effects, and residuals (Table 2). For the social genetic effects model, we obtained a significant social genetic variance of 7.4 ± 2.6, indicating the presence of SGE. The estimate of the genetic correlation between DGE and SGE was equal to − 0.24 (0.13), which indicated competitive behavior between the pigs. The estimated heritability for ADG was equal to 0.22 ± 0.01 with the classical model and the estimate of T^2^ for the social genetic effects model was surprisingly similar (0.23 ± 0.01). Nevertheless, the presence of SGE was supported both by the significant social genetic variance and by a log-likelihood test that showed that the social genetic effects model could not be reduced to the classical animal model (P < 0.05).

**Table 2:** Parameter estimates (standard errors in brackets) from a univariate classical and a social genetic effects model for average daily gain (g/day) across boars and gilts

Component	Classical	Social
${\upsigma}_{{{\mathbf{A}}_{\text{D}} }}^{2}$	2550.1 (159.5)	2521.4 (158.6)
${\upsigma}_{{{\mathbf{A}}_{\text{S}} }}^{2}$		7.4 (2.6)
${\upsigma}_{{{\mathbf{A}}_{\text{DS}} }}$		− 2.49 (16.8)
${\upsigma}_{g}^{2}$	1115.9 (35.1)	1081.9 (36.9)
${\upsigma}_{l}^{2}$	727.4 (36.9)	725.3 (44.1)
${\upsigma}_{e}^{2}$	7037.6 (86.6)	7015.4 (87.9)
$h^{2} /{\text{T}}^{2}$	0.223 (0.014)	0.234 (0.014)
$r_{{{\mathbf{A}}_{\text{D}} ,{\mathbf{A}}_{\text{S}} }}$		− 0.24 (0.126)
${\upsigma}_{TBV}^{2}$		2654.4 (106.8)
$\Delta {\text{LogL}}$		8.5 (P < 0.05)

${\upsigma}_{{{\mathbf{A}}_{\text{D}} }}^{2}$ = direct genetic variance

${\upsigma}_{{{\mathbf{A}}_{\text{S}} }}^{2}$ = social genetic variance

${\upsigma}_{{{\mathbf{A}}_{\text{DS}} }}$ = direct-social covariance

${\upsigma}_{g}^{2}$ = variance of group

${\upsigma}_{l}^{2}$ = variance of litter

$${\upsigma}_{e}^{2}$$ = residual variance

*h*^2^ = heritability

T^2^ = total heritable variation

$r_{{{\mathbf{A}}_{\text{D}} ,{\mathbf{A}}_{\text{S}} }}$ = genetic correlation between direct and social genetic effect

${\upsigma}_{TBV}^{2}$ = variance of the total breeding value

$\Delta {\text{LogL}}$ = reduction in − 2Log Likelihood compared to the classical model, P values from a likelihood ratio test with 2 degrees of freedom in parentheses

### Bivariate models in which ADG of boars and gilts are different traits

In the bivariate model, the estimate of the direct genetic variance was 17% higher for boars (2883 ± 433) than for gilts (2389 ± 164) with the classical model. The residual variance was 29% higher for boars than for gilts with both models (P < 0.05). The estimate of the social genetic variance was also higher for boars (13.8 ± 5.8) than for gilts (9.3 ± 3.9) but these estimates were not statistically different. Estimates of heritabilities were 0.22 ± 0.01 for boars and 0.24 ± 0.02 for gilts using the classical model, while estimates of T^2^ were 0.32 ± 0.02 for boars and 0.27 ± 0.01 for gilts. The estimate of the genetic correlation of DGE between boars and gilts was high and favorable (0.88 ± 0.02) with both the classical and the social genetic effects models. The group variance was lower for the social genetic effects model than for the classical model for both boars and gilts. All estimates of genetic correlations involving SGE had large standard errors. The estimate of the genetic correlation between DGE and SGE was negative and stronger for gilts (− 0.22 ± 0.14) than for boars (− 0.04 ± 0.16) but due to their large standard errors, they did not differ significantly from zero. The estimate of the genetic correlation of SGE between the two sexes was low (0.30 ± 0.30). The estimate of the genetic correlation between the DGE for boars and the SGE for gilts (− 0.22 ± 0.14) and of the genetic correlation between the DGE for gilts and the SGE for boars (− 0.064 ± 0.15) were not statistically different from 0.

### Predictions

Table 4 provides the predictive abilities (correlations of predicted breeding values with corrected phenotypes) for the classical and social genetic effects univariate and bivariate models. With the univariate model, the predictive ability of DGE was similar for the classical and social genetic models for both sexes (0.165 and 0.164). In general, predictive abilities were lower for boars than for gilts for all models. With the univariate model, predictive abilities of the sum of the social breeding values for group mates $(\sum SBV_{{SOCIAL_{j} }} )$ were equal to 0.085 and 0.067 for gilts and boars, respectively. By applying a social genetic effects model instead of a classical model, predictive ability improved from 0.165 to 0.177 for $P_{{TOTAL_{i} }}$ for boars and from 0.235 to 0.250 for gilts. This increase in predictive ability was, however, only significant (P = 0.004) for gilts. Fitting a bivariate model increased the predictive ability of the sum of the social breeding values for group mates $(\sum SBV_{{SOCIAL_{j} }} )$ considerably for boars (0.165). The predictive ability of DGE with the classical model was 0.170 for boars, which increased to 0.196 for the total phenotype with the social genetic effects model. For gilts, the predictive ability of DGE increased from 0.251 with the classical model to 0.262 with the social genetic model. With the bivariate model, the increase in predictive ability, when applying the social genetic effects model instead of the classical model was significant for both boars (P = 0.001) and gilts (P = 0.047).

## Discussion

### Genetic parameters for DGE and SGE

In this study, we estimated (co)-variance components for DGE and SGE for boars and gilts by using a bivariate model, which to our knowledge has not been previously reported. Although all estimates involving social genetic effects had large standard errors, our results indicate that both the variance of social genetic effects and the co-variances between DGE and SGE differed between gilts and boars. We estimated a genetic correlation of 0.88 between ADG in gilts and boars, which was significantly different from 1 and shows that ADG in boars and sows are different traits. This result is in contrast to estimates reported by Saintilan et al. [18], who found that genetic correlations between gilts and boars for daily gain were not significantly different from 1 (0.95), but their estimates were based on a much smaller number of observations (1121 males and 508 females).

Several reasons may explain why genetic parameters differ between sexes. First, there is a scale effect because of a difference in ADG levels between boars and gilts, which is indeed reflected in the direct variances, which were much higher for boars than for gilts. Second, there may be selection bias, since approximately 6% of the tested boars were not included in the data because they were performance-tested at a boar test station. These boars most likely belong in the upper end of the population with respect to performance for ADG but this was not reflected in lower variance for DGE for boars. Finally, there may be a biological explanation for the difference in parameter estimates between boars and gilts, since social breeding values are associated with behavioral differences. For example, pigs with low breeding values for SGE perform more non-reciprocal biting than pigs with high SGE breeding values [19]. However, no previous studies have investigated the effect of SGE breeding values on the behavior of boars and gilts when they are housed separately, since gilts and castrated males were mixed in pens in the study of Camerlink et al. [19]. Still, it has been reported that boars and gilts have different behaviors. Our estimates of the correlations between DGE and SGE indicate competitive behavior between gilts but not between the boars. Thus, more competitive behavior such as aggressions among gilts than among boars is expected. In slaughter pigs, Boyle and Björklund [20] showed that, in pens that included intact boars, antagonistic behaviors (mounting and nudging) were more frequent than in pens with gilts only. Similarly, Teixeira and Boyle [12] found that aggressive and mounting behaviors were more frequent in intact boars than in gilts. In addition, boars had more skin lesions and bruises on their carcass than gilts. Bünger et al. [13] reported higher frequencies of knocking, fighting, and mounting in intact boars than in gilts. In addition, some studies indicated that tail damage is more severe in gilts than in boars. For example, Zonderland et al. [10] investigated the number of days before 40% of the piglets had tail damage and the number of days a piglet experienced tail damage in male and females piglets housed in separate pens. In the female piglet pens, 40% of the piglets had tail damage after 11 days, whereas 40% of the male piglets had tail damage after 16 days. The duration of tail damage was also longer in the female compared to the male pens. Overall, these studies of behavioral differences between the sexes imply that boars are more competitive than gilts. However, we need more studies and data to quantify how the social genetic parameters for each sex are reflected in social behavior.

Our estimates of social genetic variances were significant for both the univariate model and the bivariate model, which indicates presence of SGE. The presence of SGE was also supported by the log likelihood test, which showed that the univariate social genetic effects model fitted the data better than the classical model. Estimates of T^2^ (0.32 for boars and 0.27 for gilts) were higher with the bivariate model than with the univariate model (0.23), which was primarily due to differences in the estimates of social genetic variances, which were higher for both boars and gilts in the bivariate (Table 3) than in the univariate model (Table 2). For the univariate model, the estimate of T^2^ was not much higher than the estimate of *h*^2^ (0.23 vs. 0.22) but this was primarily caused by the unfavorable genetic correlation observed between DGE and SGE, which reduced T^2^ [4]. This was also reflected in the results from the bivariate model, where the estimate of T^2^ increased compared to *h*^2^, especially for boars, for which the estimate of the genetic correlation between DGE and SGE was close to zero. Therefore, a relatively low T^2^ compared to *h*^2^ should not be confused with SGE being unimportant. Instead, in those cases, realized selection response to selection on DGE without considering SGE may be lower than expected based on the estimate of *h*^2^ [4]. Estimates of T^2^ were in the range of those reported in previous studies. Bergsma et al. [21] found estimates of 0.24 for *h*^2^ and of 0.34 for T^2^ for daily gain, whereas the estimate of T^2^ by Chen et al. [2] was higher, at 0.59.

**Table 3:** Parameter estimates (standard errors in brackets) from bivariate classical and a social genetic effects models for average daily gain (g/day) in gilts and boars as separate traits

Component	Model			
	Classical		Social	
	Boars	Gilts	Boars	Gilts
${\upsigma}_{{{\mathbf{A}}_{\text{D}} }}^{2}$	2883.4 (232.7)	2389.2 (163.9)	2868.9 (233.3)	2508.7 (164.5)
${\upsigma}_{{{\mathbf{A}}_{\text{S}} }}^{2}$			13.8 (5.8)	9.3 (3.9)
${\upsigma}_{{{\mathbf{A}}_{\text{DS}} }}$			− 7.58 (31.1)	− 34.0 (20.8)
${\upsigma}_{g}^{2}$	1111.0 (64.2)	1038.0 (42.1)	979.0 (87.6)	989.3 (57.4)
${\upsigma}_{l}^{2}$	930.9 (75.7)	724.1 (44.8)	927.3 (75.6)	722.5 (44.8)
${\upsigma}_{{{\mathbf{A}}_{\text{Db}} {\mathbf{A}}_{\text{Dg}} }}$			2368.6 (163.4)	
${\upsigma}_{{{\mathbf{A}}_{\text{Db}} {\mathbf{A}}_{\text{Sg}} }}$			− 35.65 (23.42)	
${\upsigma}_{{{\mathbf{A}}_{\text{Dg}} {\mathbf{A}}_{\text{Sb}} }}$			− 11.9 (28.2)	
${\upsigma}_{{{\mathbf{A}}_{\text{Sb}} {\mathbf{A}}_{\text{Sg}} }}$			3.45 (3.5)	
${\upsigma}_{{l_{{{\text{b}},{\text{g}}}} }}$	657.0 (44.5)		654.5 (44.5)	
${\upsigma}_{e}^{2}$	8270.0 (141.6)	5903.4 (92.6)	8263.8 (142.8)	5877.6 (94.1)
$h^{2} /{\text{T}}^{2}$	0.219 (0.012)	0.238 (0.016)	0.320 (0.020)	0.273 (0.014)
${\upsigma}_{TBV}^{2}$			4286.2 (263.76)	2795.2 (140.2)
$r_{{{\mathbf{A}}_{\text{Db}} ,{\mathbf{A}}_{\text{Dg}} }}$	0.883 (0.02)		0.882 (0.02)	
$r_{{{\mathbf{A}}_{\text{D}} ,{\mathbf{A}}_{\text{S}} }}$			− 0.038 (0.157)	− 0.222 (0.140)
$r_{{{\mathbf{A}}_{\text{Sb}} ,{\mathbf{A}}_{\text{Sg}} }}$			0.304 (0.304)	
$r_{{{\mathbf{A}}_{\text{Db}} ,{\mathbf{A}}_{\text{Sg}} }}$			− 0.218 (0.144)	
$r_{{{\mathbf{A}}_{\text{Dg}} ,{\mathbf{A}}_{\text{Sb}} }}$			− 0.064 (0.152)	
$\Delta {\text{LogL}}$			10.3 (P = 0.17)	

${\upsigma}_{{{\mathbf{A}}_{\text{D}} }}^{2}$ = direct genetic variance

${\upsigma}_{{{\mathbf{A}}_{\text{S}} }}^{2}$ = social genetic variance

${\upsigma}_{{{\mathbf{A}}_{\text{DS}} }}$ = direct-social covariance

${\upsigma}_{g}^{2}$ = variance of group

${\upsigma}_{l}^{2}$ = variance of litter

$${\upsigma}_{e}^{2}$$ = residual variance

${\upsigma}_{{{\mathbf{A}}_{\text{Db}} {\mathbf{A}}_{\text{Dg}} }}$ = covariance between direct effects in boars and gilts

${\upsigma}_{{{\mathbf{A}}_{\text{Db}} {\mathbf{A}}_{\text{Sg}} }}$ = covariance between direct effects in boars and social effects in gilts

${\upsigma}_{{{\mathbf{A}}_{\text{Dg}} {\mathbf{A}}_{\text{Sb}} }}$ = covariance between direct effects in gilts and social effects in boars

${\upsigma}_{{{\mathbf{A}}_{\text{Sb}} {\mathbf{A}}_{\text{Sg}} }}$ = covariance between social effects in boars and gilts

*h*^2^ = heritability

T^2^ = total heritable variation

${\upsigma}_{TBV}^{2}$ = variance of the total breeding value

$r_{{{\mathbf{A}}_{\text{Db}} ,{\mathbf{A}}_{\text{Dg}} }}$ = genetic correlation between direct effects in boars and gilts

$r_{{{\mathbf{A}}_{\text{D}} ,{\mathbf{A}}_{\text{S}} }}$ = genetic correlation between direct and social genetic effects

$r_{{{\mathbf{A}}_{\text{Sb}} ,{\mathbf{A}}_{\text{Sg}} }}$ = genetic correlation between social effects in boars and gilts

$r_{{{\mathbf{A}}_{\text{Db}} ,{\mathbf{A}}_{\text{Sg}} }}$ = genetic correlation between direct effects for boars and social for gilts

$r_{{{\mathbf{A}}_{\text{Dg}} ,{\mathbf{A}}_{\text{Sb}} }}$ = genetic correlation between direct effects for gilts and social for boars

$\Delta {\text{LogL}}$ = reduction in − 2Log Likelihood compared to the classical model

P values from a likelihood ratio test with 7 degrees of freedom in parentheses

### Differences between the univariate and bivariate models

Our results weakly indicate that the predictive ability for both boars and gilts increased by applying a bivariate instead of a univariate model (Table 4). Also the correlation of $DBV_{{CLASSIC_{i} }}$ with corrected phenotypes increased for the bivariate model, indicating that even a model without social genetic effects, the bivariate model is more appropriate than the univariate model. This was also supported by the fact that the estimate of the genetic correlation between ADG in boars versus gilts was significantly less than 1 (0.88) (Table 3). When applying the univariate classical and social genetic effects models, genetic parameter estimates (variance of DGE and litter effects, and the correlation between DGE and SGE) were similar to those obtained for gilts using the bivariate model. As a result, in particular the predictive ability for boars increased when the bivariate instead of the univariate model was used. This was also supported by the greater increase in predictions of $\sum SBV_{{SOCIAL_{j} }}$ for boars than for sows when the bivariate instead of the univariate social genetic effects model was used. Finally, the increase in predictive ability from using a social genetic effects model compared to a classical model using the bivariate model was significant for both boars (*P* = 0.047) and gilts (*P* = 0.001). In the univariate model, the increase in predictive ability was not significant for boars.

**Table 4:** Pearson correlations (standard errors in brackets) of predicted breeding values and corrected phenotypes for the univariate and the bivariate model based on 431 boars and 876 gilts

Model	Corrected phenotype^a^	Predicted breeding values				
		$DBV_{{CLASSIC_{i} }}$	$DBV_{{SOCIAL_{i} }}$	$\sum SBV_{{SOCIAL_{j} }}$	$P_{{TOTAL_{i} }}$	P value^b^
*Univariate*						
Boars	$\hat{P}_{iu}$	0.165 (0.047)	0.164 (0.047)	0.067 (0.048)	0.177 (0.047)	0.15
Gilts	$\hat{P}_{iu}$	0.235 (0.033)	0.235 (0.033)	0.085 (0.034)	0.250 (0.032)	0.004
*Bivariate*						
Boars	$\hat{P}_{ib}$	0.170 (0.047)	0.169 (0.048)	0.165 (0.047)	0.196 (0.047)	0.001
Gilts	$\hat{P}_{ib}$	0.251 (0.033)	0.251 (0.033)	0.069 (0.034)	0.262 (0.033)	0.047

$DBV_{{CLASSIC_{i} }}$ = direct breeding value for individual *i* from the classical model

$DBV_{{SOCIAL_{i} }}$ = direct breeding value from individual *i* from the social genetic model

$\sum SBV_{{SOCIAL_{j} }}$ = sum of the social breeding values of mates *j*

$P_{{TOTAL_{i} }}$ = total phenotype of an individual calculated as: $P_{{TOTAL_{i} }} = DBV_{{SOCIAL_{i} }} + \sum SBV_{{SOCIAL_{j} }}$

^a^Corrected phenotypes from univariate (u) and bivariate (b) models, respectively

^b^P value tests if the correlation between the corrected phenotypes and $DBV_{{CLASSIC_{i} }}$ is statistically different from the correlation between the corrected phenotypes and $P_{{TOTAL_{i} }}$

### Group composition

Bijma [8] showed that the optimal design for estimating direct and social genetic parameters is one in which groups consist of animals from two families. He also showed that 250 to 500 groups are needed in order to accurately estimate variance components [8]. Especially, the correlation between DGE and SGE is difficult to estimate accurately [22]. In our study, we had 7309 groups. However, since our data came from a herd performance test program and not from an experiment, our groups were composed of varying numbers and sizes of families. Group sizes were also relatively large, which means that they were always composed of more than two families. Thus, the group composition in our data was probably not optimal for estimation of variance components. However, in practical pig breeding, it is unrealistic to perform an experiment with 500 groups of two families each. Another complicating factor is that our pigs were housed in different breeding herds, which makes it more difficult to estimate SGE due to differences in the environment between herds [23]. The environment of the herds was standardized with respect to feeding, density, etc., but herds differed in how pigs were mixed prior to the performance test. In some herds, pigs were less likely to be mixed with other pigs from the same litter than in other herds. This may be a problem because the phenotypic social effects of a pig depends on how much it is related to the recipient [23]. Recently, Canario et al. [24] showed that early-life social effects in pigs affect the growth of their group mates later in life, such that pigs that have been through the same early life experience, develop similar social skills. Such effects may have an impact on our estimates of SGE because some pigs were more likely to be mixed with pigs from their own litter than with pigs from other litters. Moreover, Alemu et al. [25] showed that the social genetic model may result in biased estimates of social genetic parameters if the groups consist of a combination of sibs and random individuals. This can be solved partly by dividing effects into SGE on kins and SGE on unrelated individuals [23, 25]. However, when SGE differ between kins and non-kins, genetic parameters are not fully identifiable because of confounding between direct and social effects of members of the same families [25]. We investigated fitting the number of sibs in a group and the genetic relationship within a group as covariates in the model, but this did not improve the predictability of our models, and thus they were not included in the final models.

The increase in predictive ability from using a social genetic effects model compared to a classical animal model was only significant for gilts in the univariate model. Duijvesteijn [6] showed that the accuracy of social breeding values is mainly determined by the number of sires represented in both the prediction and the validation dataset. The improvement in predictive ability from using a social genetic model compared to a classical model was not significant [6]. The study by Duijvesteijn [6] included data on progeny from 68 sires, whereas we had only 45 sires. It is difficult to increase the number of sires because sires must have offspring in both the prediction and validation datasets and because the prediction must be performed in whole groups [6]. Adding genomic information may be a way to increase the accuracy of predictions of breeding values. Alemu et al. [26] showed that the accuracy increased by 32 and 35% when applying genomic predictions in a sire model including SGE in two lines of laying hens compared to using parent average estimated breeding values.

The pigs in our study were grouped in pens of different sizes. The SGE may depend on group size, which can be accounted for by adding a dilution effect [27]. In our initial analyses, we investigated fitting a dilution effect in the univariate social genetic effects model. However, since including a dilution effect did not increase the fit of the model, it was not included in the final models.

### Practical implications

We used data on purebred gilts and boars, but in practice, slaughter pigs are crossbreds. Ellen et al. [5] discussed genetic improvement for traits affected by social interactions in relation to crossbred performance. If the correlation between purebred and crossbred performance is low, information from crossbreds is needed to improve socially affected traits, as is the case with the DGE [28, 29]. To date, we are not aware of studies that have quantified the relationships of SGE between purebreds and crossbreds.

We used purebred gilts and boars that were kept in separate pens, but in practice, gilts and boars may be mixed. Boars and gilts will probably behave differently in mixed than in sex-specific pens. With mixed pens, it would be possible to divide SGE according to the sexes of the focal and recipient pigs [4]. To date, how the sex-specific SGE are expressed in mixed pens is unknown. In addition, when boars and gilts are selected for SGE in sex-specific pens, there is the question of which estimated breeding value should be used in selection since both boars and gilts get one estimated breeding value for each sex. Because their progeny will be half males and half females, selecting both males and females on the average estimated social breeding value for each sex would be an option.

## Conclusions

In this study, we tested the hypothesis that DGE and SGE for ADG in pigs differ between boars and gilts and that accounting for these differences will improve the predictability of the social genetic effects model. Using a bivariate social genetic effects model resulted in different variance component estimates than the univariate model. For boars, the estimate of the covariance between DGE and SGE was close to 0 but highly negative for gilts, which indicates that there is competition between gilts but not between boars. For both the classical and social genetic effects models, the estimate of the genetic correlation of ADG between gilts and boars was significantly less than 1 (0.88), which indicates that ADG in boars and gilts are different traits. Based on this result, and the fact that the bivariate social genetic effects model had better predictive ability than the univariate model, we suggest that ADG in boars and gilts should be treated as separate traits in both classical and social genetic effects models. Significant genetic variances of SGE for ADG show the presence of SGE for both the univariate and bivariate models, which indicate that a social genetic effects model should be used instead of a classical model.

## References

[1] Bergsma R, Kanis E, Knol EF, Bijma P (2008). The contribution of social effects to heritable variation in finishing traits of domestic pigs (*Sus scrofa*). Genetics.

[2] Chen CY, Kachman SD, Johnson RK, Newman S, Van Vleck LD (2008). Estimation of genetic parameters for average daily gain using models with competition effects. J Anim Sci.

[3] Canario L, Lundeheim N, Bijma P. Pig growth is affected by social genetic effects and social litter effects that depend on group size. In: Proceedings of the 9th world congress on genetics applied to livestock production: 1–6 August 2010; Leipzig. 2010.

[4] Bijma P, Muir WM, van Arendonk JAM (2007). Multilevel selection 1: quantitative genetics of inheritance and response to selection. Genetics.

[5] Ellen ED, Rodenburg TB, Albers GAA, Bolhuis JE, Camerlink I, Duijvesteijn N (2014). The prospect of selection for social genetic effects to improve welfare and productivity in livestock. Front Genet.

[6] Duijvesteijn N. Validation of indirect genetic effects for average daily gain in pigs. In: Sociable Swine: prospects of indirect genetic effects for the improvement of productivity, welfare and quality. PhD thesis, 2015, p. 79–96.

[7] Ellen ED, Muir WM, Teuscher F, Bijma P (2007). Genetic improvement of traits affected by interactions among individuals: sib selection schemes. Genetics.

[8] Bijma P (2010). Estimating indirect genetic effects: precision of estimates and optimum designs. Genetics.

[9] Blanchard PJ, Ellis M, Warkup CC, Chadwick JP, Willis MB (1999). The influence of sex (boars and gilts) on growth, carcass and pork eating quality characteristics. Anim Sci.

[10] Zonderland JJ, Bracke MBM, den Hartog LA, Kemp B, Spoolder HAM (2010). Gender effects on tail damage development in single-or mixed-sex groups of weaned pigs. Livest Sci.

[11] Kritas SK, Morrison RB (2007). Relationships between tail biting in pigs and disease condemnations at slaughter. Vet Rec.

[12] Teixeira DL, Boyle LA (2014). A comparison of the impact of behaviours performed by entire male and female pigs prior to slaughter on skin lesion scores of the carcass. Livest Sci.

[13] Bünger B, Schrader L, Schrade H, Zacharias B (2015). Agonistic behaviour, skin lesions and activity pattern of entire male, female and castrated male finishing pigs. Appl Anim Behav Sci.

[14] Muir WM (2005). Incorporation of competitive effects in forest tree or animal breeding programs. Genetics.

[15] Bijma P, Muir WM, Ellen ED, Wolf JB, van Arendonk JAM (2007). Multilevel selection 2: estimating the genetic parameters determining inheritance and response to selection. Genetics.

[16] Madsen P, Jensen J. A User’s Guide to DMU. A package for analyzing multivariate mixed models. 2013. http://dmu.agrsci.dk/DMU/Doc/Current/dmuv6_guide.5.2.pdf. Accessed 7 July 2017.

[17] Steiger JH (1980). Tests for comparing elements of a correlation matrix. Psychol Bull.

[18] Saintilan R, Sellier P, Billon Y, Gilbert H (2012). Genetic correlations between males, females and castrates for residual feed intake, feed conversion ratio, growth rate and carcass composition traits in Large White growing pigs. J Anim Breed Genet.

[19] Camerlink I, Turner SP, Bijma P, Bolhuis E (2013). Indirect genetic effects and housing conditions in relation to aggressive behavior in pigs. PLoS One.

[20] Boyle LA, Björklund L (2007). Effects of fattening boars in mixed or single sex groups and split marketing on pig welfare. Anim Welf.

[21] Bergsma R, Mathur PK, Kanis E, Verstegen MWA, Knol EF, van Arendonk JAM (2013). Genetic correlations between lactation performance and growing-finishing traits in pigs. J Anim Sci.

[22] Ødegård J, Olesen I (2011). Comparison of testing designs for genetic evaluation of social effects in aquaculture species. Aquaculture.

[23] Bijma P (2013). The quantitative genetics of indirect genetic effects: a selective review of modelling issues. Heredity (Edinb).

[24] Canario L, Lundeheim N, Bijma P (2017). The early-life environment of a pig shapes the phenotypes of its social partners in adulthood. Heredity (Edinb).

[25] Alemu SW, Berg P, Janss L, Bijma P (2014). Indirect genetic effects and kin recognition: estimating IGEs when interactions differ between kin and strangers. Heredity (Edinb).

[26] Alemu SW, Calus MPL, Muir WM, Peeters K, Vereijken A, Bijma P (2016). Genomic prediction of survival time in a population of brown laying hens showing cannibalistic behavior. Genet Sel Evol.

[27] Bijma P (2010). Multilevel selection 4: modeling the relationship of indirect genetic effects and group size. Genetics.

[28] Wei M, van der Werf J (1994). Maximizing genetic response in crossbreds using both purebred and crossbred information. Anim Sci.

[29] Bijma P, van Arendonk JAM (1998). Maximizing genetic gain for the sire line of a crossbreeding scheme utilizing both purebred and crossbred information. Anim Sci..

